# Comparison of Sample Preparation Techniques for Inspection of Leaf Epidermises Using Light Microscopy and Scanning Electronic Microscopy

**DOI:** 10.3389/fpls.2020.00133

**Published:** 2020-02-25

**Authors:** Jinhong Yuan, Xiaoduan Wang, Huihui Zhou, Yulin Li, Jing Zhang, Shuxin Yu, Mengni Wang, Menghan Hao, Qian Zhao, Le Liu, Mingjun Li, Junhua Li

**Affiliations:** Engineering Technology Research Center of Nursing and Utilization of Genuine Chinese Crude Drugs, College of Life Sciences, Henan Normal University, Xinxiang, China

**Keywords:** leaf epidermis, light microscope, laser scanning confocal microscope, scanning electronic microscope, Coolstage

## Abstract

The micro-morphology of leaf epidermises is valuable for the study of leaf development and function, as well as the classification of plant species. There have been few studies comparing different preparation and imaging methods for visualizing the leaf epidermis. Here, four specimen preparation methods were used to investigate the leaf epidermis morphology of *Arabidopsis*, radish, cucumber, wheat, rice, and maize, under an inverted basic light microscope (LM), a laser scanning confocal microscope (LSCM), or a scanning electron microscope (SEM). Optical microscope specimens were obtained using either the direct isolation method or the chloral hydrate-based clearing method. SEM images were obtained using a standard stage for conventional dehydrated samples or a Coolstage for fresh tissue. Different parts of epidermis peels were well focused under the LM. Investigation of samples cleared by chloral hydrate is convenient and autofluorescence of cell walls can be detected in rice. The resolution of images of conventional SEM leaf samples was generally higher than the Coolstage images at the same magnification, whereas local collapse and shrinkage were observed in leaves with high water content when using the conventional method. However, stomatal apparatuses of *Arabidopsis*, cucumber, radish, and maize deformed and showed poor appearance when using the Coolstage. Moreover, we usually used glutaraldehyde as an SEM fixative when using t-butanol for freeze-drying, though methanol is considered a better fixative in recent studies. In addition, fresh samples were not stable on the Coolstage. Thus, we compared four different t-butanol freeze-drying methods and two Coolstage methods. The dimension and morphology of tissues were compared using the six different methods. The results indicate that methanol fixative obviously reduced shrinkage of SEM samples compared with glutaraldehyde and formaldehyde alcohol acetic acid (FAA) fixatives. The use of methanol and a graded series of steps improved the preservation of samples. Preparing samples with optimal cutting temperature compound and observing at −30°C helped to increase the stability of Coolstage samples. In summary, our results provide an overview of the shortcomings and merits of four different methods, and might provide some information about choosing an optimal method for visualizing epidermal morphology.

## Introduction

The leaf is the primary site of organic compound production and gas exchange. Observing the micro-structure of the leaf epidermis is of important theoretical and applied value. For example, observation of the micro-structure of glandular hairs contributes to understanding of their ontogeny, anatomy, secretory compound composition, and relationship to herbivores (Nery et al., 2017; Yan et al., 2017). Moreover, analysis of epidermis micro-structure can provide evidence for taxonomic classification and genetic relationships (Kang et al., 2017; Khan et al., 2017; Gutiérrez-Ortega et al., 2018; Hussain et al., 2018; Shah et al., 2018).

In most studies, a light microscope (LM) or a scanning electronic microscope (SEM) is used to investigate the micro-morphology of leaf epidermises. In light microscopy, transmission light is generally used as the light source. In order to observe leaf micro-morphology, a large number of studies have been conducted to investigate leaf epidermis (Green and Linstead, 1990; Schulte et al., 2009; Bailes and Glover, 2018). Because leaf blades are thick and contain a large amount of chlorophylls, focusing on the epidermis of an intact blade under an LM is difficult, and it is hard to distinguish the cell outlines from the background. One solution is to separate the epidermis from the mesophyll tissue during sample preparation using methods such as direct isolation (by peeling or scraping), peeling after boiling in maceration agents, for example, chromic acid/nitric acid (Stockey et al., 1995; Shah et al., 2018). Another method for visualizing the epidermis is to prepare transparent leaf tissues using clearing agents such as KOH, NaOH, hypochlorite, and chloral hydrate (Morawetz, 2013; Shah et al., 2018; Vovides et al., 2018). For example, Cheng et al. (2014) developed a method for clearing leaf blades and successfully applied it to *Arabidopsis* (*Arabidopsis thaliana*). The leaf tissues were decolorized with 70% ethanol and cleared with a solution containing chloral hydrate. There is no need to peel the epidermis using this method, and both the adaxial and abaxial epidermises can be observed simultaneously. Besides, there are other methods that have been developed to investigate leaf micro-morphology, including making impressions of the leaf epidermis with clear fingernail polish (Waisel et al., 1969; Gitz and Baker, 2009).

SEM images are produced by bombarding the sample's surface with a focused beam of electrons, which releases electrons with lower energies. An electron beam has a much shorter wavelength than that of visible light of an optical microscope. Thus, compared with LMs, both the resolution and magnification of SEMs are greatly improved. Previous studies often used a graded series of ethanol or tertiary butanol (t-butanol) for SEM sample preparation, since using a series of solvents might produce a better effect than using just one grade. However, the treatment using a chemical gradient is time-consuming. Conventional SEM samples need to be treated with a series of procedures: fixed in chemicals first, and then gradually dehydrated with ethanol, followed by substituting ethanol with other organic solvents such as acetone, isoamyl acetate, or t-butanol (Inoué and Osatake, 1988; Wang et al., 2000; Pathan et al., 2010); and finally dried using the critical point drying (CPD) or t-butanol freeze-drying method (Inoué and Osatake, 1988; Pathan et al., 2010). It can be problematic because the leaves of many plants are soft and tender and have high water contents. In addition, special instruments are often required to dry the specimens and to coat them with metal. This metal-coating step is necessary to avoid the accumulation of electron charges on sample surfaces, which are non-conductive. Numerous studies have been reported over the years and methods have been modified to become simpler, faster, and produce better results (Neinhuis and Edelmann, 1996; Talbot and White, 2013a; Talbot and White, 2013b; Baskin et al., 2014). Although much progress has been made, the conventional sample preparation procedure is complicated and time consuming, and might induce the shrinkage and collapse of epidermis cells.

Another approach is to observe the fresh epidermis directly using SEMs such as an SEM equipped with a Coolstage, an environmental SEM (Muscariello et al., 2005; McCully et al., 2009; Mestres et al., 2011), or a cryo-SEM (Read and Jeffree, 1991; Choi et al., 2016). Specimens observed under a cryo-SEM were found to be stable under ultra-low temperature and did not easily accumulate electrostatic charges (Nijsse and van Aelst, 1999; McCully et al., 2009). The quality of the images was better than that of images obtained from an SEM equipped with a Coolstage or an environmental SEM (McCully et al., 2009; Mestres et al., 2011). However, cryo-SEMs are expensive and require a high level of technical expertise; therefore, they are still not widely used.

When investigating the micro-morphology of the leaf epidermis, the choice of whether to use an LM or SEM will affect imaging because different methods are used for sample illumination. It is hard for a researcher who is not familiar with different kinds of microscopes [i.e., LM, laser scanning confocal microscope (LSCM), and SEM] to choose a suitable method. Moreover, highly equipped microscopes such as LSCM and SEM are more expensive than basic LMs and may not be an option for some researchers. The choice of specimen preparation method will also affect the quality of leaf sample images. It is time-consuming to try all preparation methods and microscopes for each application. A comparison of LM, LSCM, and SEM images obtained using different specimen preparation and imaging methods would allow suitable methods to be chosen based on the experimental objective and equipment available, which could save money and improve work efficiency. Although several studies have compared different specimen preparation and imaging methods using an SEM or an LM (Uwins et al., 1993; Choi et al., 2016; Urban et al., 2018), there remains a lack of studies focused on choosing a suitable method and microscope for visualizing epidermis morphology.

In order to provide some information for investigation of the micro-morphology and appearance of leaf epidermises, four types of preparation methods using different microscopes were selected and compared. The first method is to directly isolate the epidermis from the leaf. The second is to produce a transparent leaf sample. The third is to prepare a dehydrated SEM sample. The last is to observe a fresh sample on an SEM. We used the leaves of *Arabidopsis* (*Arabidopsis thaliana* L.), radish (*Raphanus sativus* L.), cucumber (*Cucumis sativus* L.), wheat (*Triticum aestivum* L.), rice (*Oryza sativa* L.), and maize (*Zea mays* L.) as materials. For LM investigation, the use of the chloral hydrate-based clearing method to prepare transparent leaf blades was compared with the direct isolation method in which epidermises were directly peeled or scraped off from leaf blades. The performance of LMs, including an inverted basic LM and an LSCM, was compared with that of the SEMs. Both conventional SEM preparation method and the method using fresh samples under an SEM equipped with a Coolstage were used in this study. Based on our findings, we discuss the merits and shortcomings of these four preparation and imaging methods to observe the leaf epidermises in these varieties. Furthermore, we compared six different SEM methods in relation to their effect on tissue dimension and morphology. The improved method is also discussed here.

## Materials and Methods

### Plant Materials

Radish and maize were grown in the field, and cucumber, wheat, and rice were grown in pots that were placed under natural condition. The average day/night temperatures were 25/16°C during the experiment. The cucumber and rice plants used for fluorescence investigation were grown in a Percival plant incubator set at a 14 h light (28°C)/8 h dark (18°C) cycle, < 75% relative humidity (RH), and a light intensity of 450 mE·s^−1^·m^−2^. *Arabidopsis* plants were grown in a growth room set at a constant temperature of 22°C, a 14 h light/10 h dark cycle, 40 to 60% RH, and a light intensity of 63 mE·s^-1^·m^-2^.

### Main Equipment

SEM (HITACHI TM3030Plus, Japan), equipped with a Peltier stage (Deben MK3 Coolstage, UK). Freeze dryer (Christ Alpha1-2LDplus, Germany). Ion Sputtering Instrument (KYKY SBC-12, China). Inverted LM (Leica DMIL, Germany) equipped with a digital color camera (Leica DFC450C, Germany). Inverted LSCM (Leica TCS SP8, Germany).

### The Preparation of Leaf Samples for Observation Under an LM or an LSCM Epidermises Obtained by Direct Isolation

It is common to prepare leaf epidermis specimens by direct isolation. The leaf epidermises of *Arabidopsis*, cucumber, and radish were directly peeled off from the leaf blades with tweezers. The epidermises of rice, maize, and wheat leaves were obtained by using blades to scrape off the mesophyll cells from the epidermis. The epidermis was spread flat in water, pressed slightly with a cover glass, and then observed under an inverted microscope or an LSCM. Most studies were conducted on the abaxial epidermis; in radish, we conducted the experiment on the adaxial epidermis.

### Preparation of Transparent Leaf Blades by Chloral Hydrate Clearing

#### Sampling

The leaf blade was cut into small pieces (3∼5 mm^2^) while avoiding the main leaf veins.

#### Treatment

*Arabidopsis* leaves were treated as described previously (Cheng et al., 2014). For leaves of other plants, some modifications were made. To extend the storage period, leaf samples were fixed in 2.5% glutaraldehyde (in phosphate buffer) or formaldehyde-acetic acid-ethanol (5%:6%:45%) fixative before treatment. Moreover, the treatment time for the leaves of other plants was extended by several days to allow complete decolorization and clearing. It was suggested that chloral-hydrate cleared tissue can be stored for extended periods in 50% glycerol containing a low concentration of sodium azide to prevent bacterial or fungal contamination. Additionally, gentle to strong heating and/or a brief bleaching treatment (4% household bleach) can generally reduce clearing times markedly (Morawetz, 2013).

#### Observation

For differential interference contrast (DIC) imaging, we used the LSCM (Leica TCS SP8, Germany) instrument to take bright-field images as other microscopes available in our department did not have a DIC module. The lightpath in the LSCM is the same as in a conventional bright-field microscope except with a laser as the light source. The LSCM is equipped with a high configured objective lens (APO level) and a DIC module, which might produce images with high resolution and a pseudo 3D effect. However, the detector (TLD) collecting transmitted light on the LSCM might be inferior to a highly equipped digital camera on a compound microscope. For visualization of the autofluorescence of fresh tissues, pieces of *Arabidopsis*, cucumber, and rice were separately mounted and observed using an excitation/emission light at 405/430–550 nm.

### SEM Samples Prepared and Observed for Comparison with LM

#### SEM Coolstage Method

Fresh leaf tissues were observed using the SEM equipped with the Coolstage, which maintained the tissues at temperatures below 0°C to avoid water evaporation (Nery et al., 2017; Yuan et al., 2019). Samples observed at −15°C were used to represent the SEM Coolstage method to compare with other methods. Leaves were separated from plants and put on ice to keep them fresh. The ends of rice leaves were placed into water to avoid water loss. Samples were attached to the Coolstage (kept at −15°C) with carbon sticky pads.

Moreover, we used an optimum cutting temperature (OCT) compound to prepare the fresh samples for investigation at −30°C. First, OCT compound was filled in the stub and the tissue was placed in it. Then the stub was frozen in the Coolstage set at −30°C.

#### Conventional SEM t-Butanol Freeze-Drying Method

The t-butanol freeze-drying method (Inoué and Osatake, 1988) is a conventional method that is considered to yield good samples. It was reported that t-butanol is equivalent or sometimes superior to CPD (Inoué and Osatake, 1988; Baskin et al., 2014). The t-butanol freeze-drying method is commonly used to prepare biological SEM samples in Japan (Kaneko et al., 1990; Tuji, 2000; Watanabe et al., 2015), China (Xin et al., 2012), and other regions (El Sharaby et al., 2012). Methanol is reported to be a better fixative than glutaraldehyde and FAA when preparing SEM samples (Neinhuis and Edelmann, 1996; Pathan et al., 2010; Talbot and White, 2013b). However, regardless of the shrinkage ratio, using glutaraldehyde as a fixative could also sufficiently preserve the shape of leaf epidermis samples. Moreover, samples in the glutaraldehyde solution could be stored at 4°C for an extended period of time. Thus, the method using glutaraldehyde as a fixative was used here to represent the conventional SEM t-butanol freeze-drying method. This method is named as method “Glut.” In this method, samples (3–5 mm^2^) avoiding the main leaf veins were first immersed in 0.1 M phosphate buffer solution (PBS) for 2–3 min. Then the samples were immersed in 2.5% glutaraldehyde (in PBS) and evacuated to make sure they sank. After fixation in glutaraldehyde overnight, water in the specimens is replaced with a graded series of ethanol (30, 50, 70, 80, 90, 100, 100%—once for 15 min on ice), and then ethanol is replaced with a graded series of t-butanol (70, 80, 90, 100, 100%—once for 15 min at each step). The t-butanol and the remaining intracellular water are finally removed by freeze-drying. The freeze-dried samples were coated with gold at 5–10 mA for 80 s, and then observed under an SEM using a standard sample stage at 5 or 15 kV accelerating voltage under a vacuum of 3–5 Pa. The SEM images were acquired at the same scanning rate at either a second electron mode or a mixed observation mode (second electron and backscattered electron).

Methanol is generally considered to be the first choice as an SEM fixative. The t-butanol freeze-drying method of Baskin et al. (2014) also used methanol as the fixative. There remains a lack of studies on the effect of different fixatives on the leaf morphology and dimension when a final freeze-drying with t-butanol is used for SEM preparation. Thus, we assessed four different methods to compare methanol with other commonly used SEM fixatives. Each of these methods included a final step of t-butanol drying. Talbot and White (2013b) developed a method using methanol as a fixative. This method is fast and produces better preservation of tissue morphology compared with the glutaraldehyde method. In this method, samples were initially fixed in methanol (10 min) and then dehydrated in ethanol twice (30 min each step) prior to CPD treatment. Here, we carried out four comparative tests of different methods. The first test was performed utilized methanol and ethanol at room temperature and was named “RT“—it included the following steps: methanol fixation (10 min) followed by ethanol dehydration (30 min, twice) at room temperature; treatment with a t-butanol series (70, 80, 90, 100, 100%, once for 15 min at each step); freeze-drying. The second test was named “Glut.” The third test was named “FAA”; the procedure of which is almost the same as method “Glut,” except that the fixative is FAA. The last test was named “Meth.” Using this method, cut samples were fixed immediately in methanol for 10 min, followed by the same steps as the “Glut” group.

### Leaf Water Content Calculation, Measurement of Tissue Area, and Statistical Analysis

Leaf samples were heat-killed in a drying oven at 110°C for 0.5 h and dried to constant mass at 70°C. The water content of leaf was calculated as follows: (1-dry weight/fresh weight) × 100%. Water content determination for each of the species was replicated five times.

Leaf tissues were imaged on a dissecting microscope (Olympus SZ61, Japan) and then transferred to fixative solution. Dried tissues were imaged again after final freeze-drying. Fresh tissues attached to a Coolstage were imaged to measure the tissue area. Areas of leaf tissues were measured following the steps outlined by Cheng et al. (2014), and 6–12 replicate leaf pieces were measured.

A randomized complete block design was used in this study. The data in the graphs were subjected to analysis of variance (ANOVA) and means were compared by Duncan’s new multiple range test at the 5% level. The data of swelling ratios and stability of Coolstage samples (min) were analyzed by t-test. All analyses were performed with version 13.0 of SPSS software. The histograms were drawn with version 10.0 of SigmaPlot software.

## Results

### Observation of Directly Isolated Leaf Epidermises Under a Basic LM

**Figure 1:**
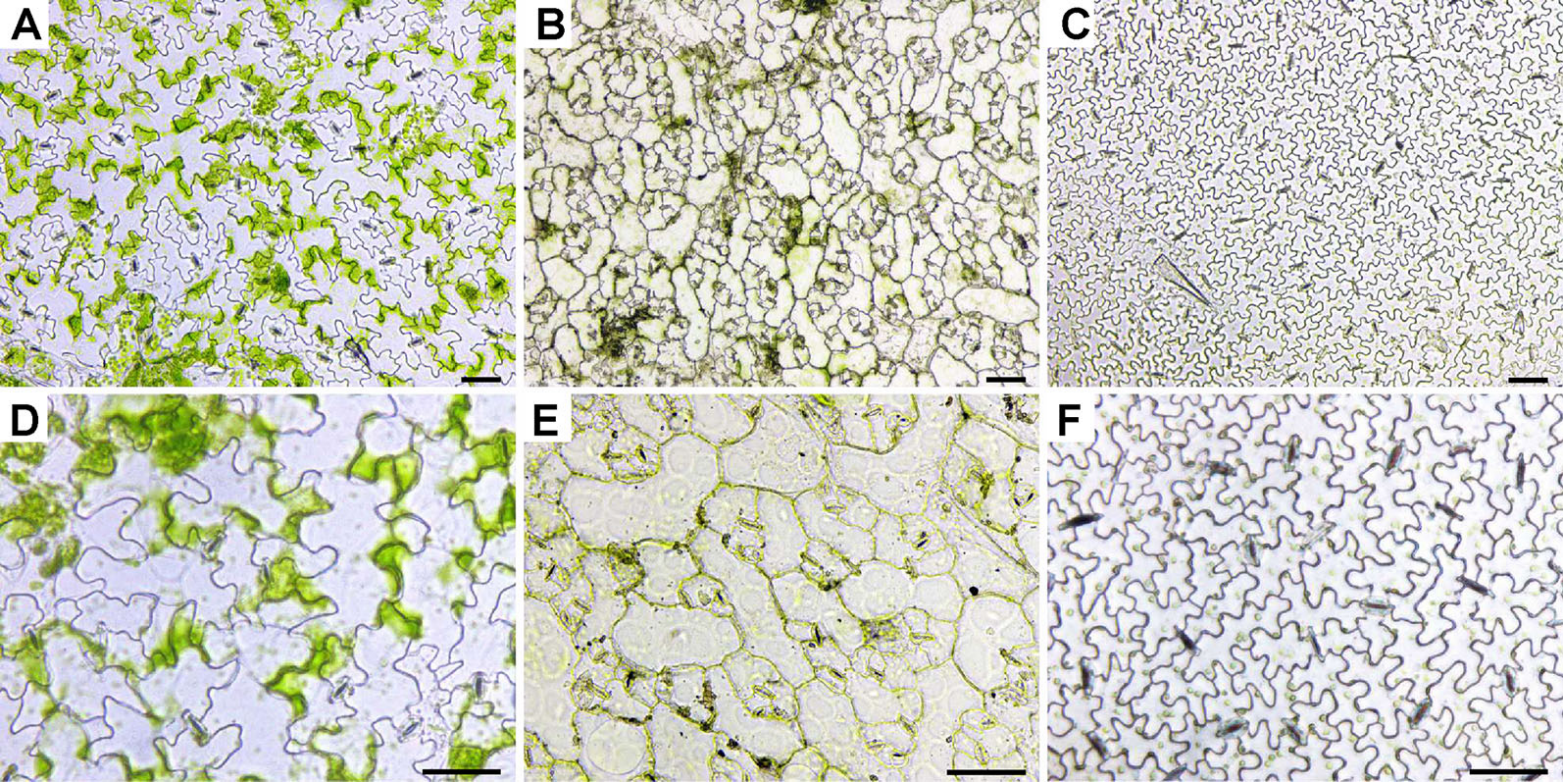
Images of leaf epidermises obtained by direct peeling taken under an inverted LM. The images were of abaxial epidermis except for radish, which was of adaxial epidermis and the same hereinafter. **(A, D)** *Arabidopsis* (*Arabidopsis thaliana* L.); **(B, E)** radish (*R. sativus* L.); **(C, F)** cucumber (*Cucumis sativus* L.). Images **(A–C)** were taken under low magnification, while images **(D–F)** were taken under high magnification. Bar=50 μm.

**Figure 2:**
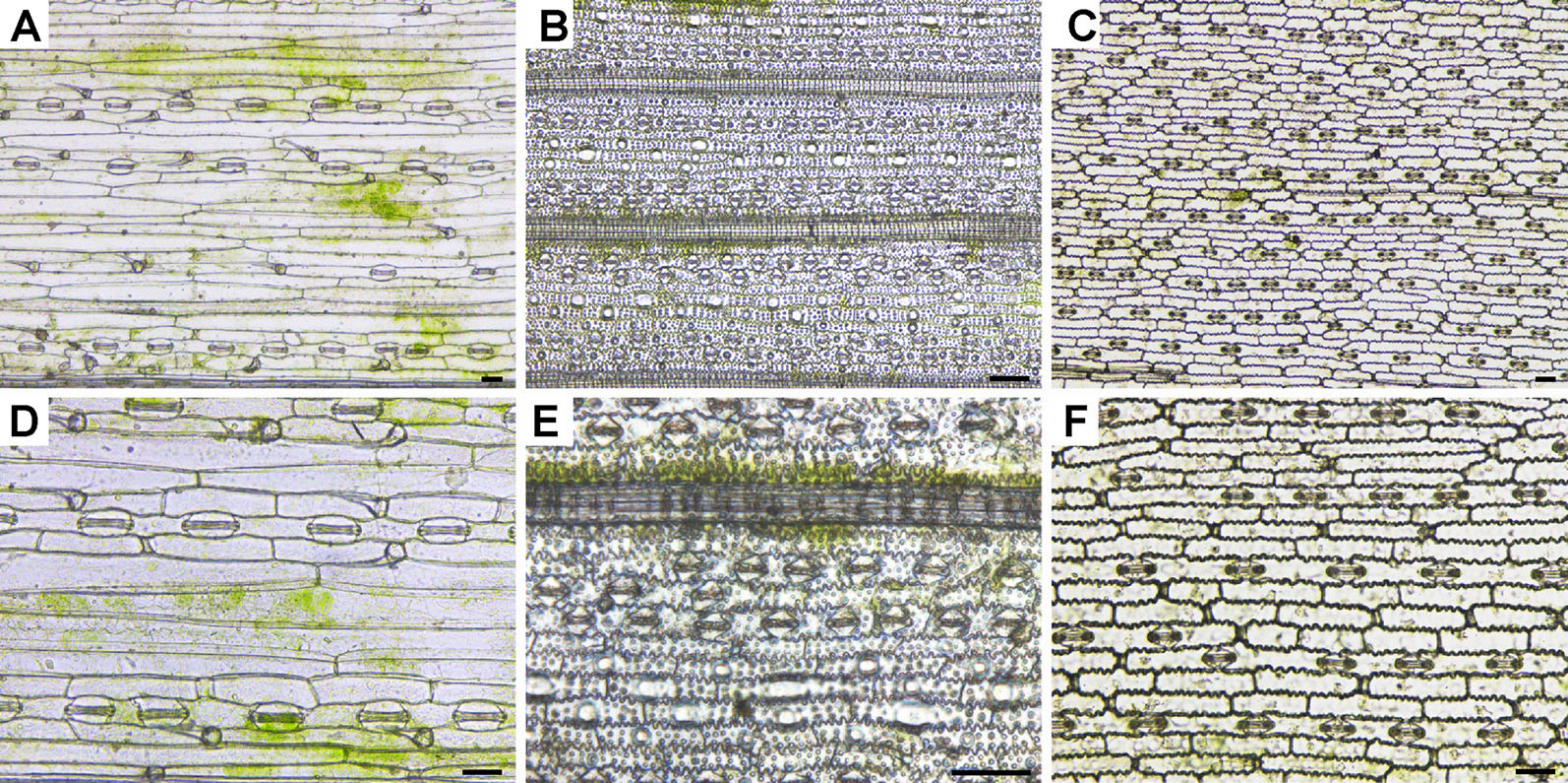
Images of leaf epidermises obtained by direct scraping taken under an inverted LM. **(A, D)** Wheat (*Triticum aestivum* L.); **(B, E)** rice (*Oryza sativa* L.); **(C, F)**, maize (*Zea mays* L.). Images **(A–C)** were taken under low magnification, while images **(D–F)** were taken under high magnification. Bar=50 μm.

Plant epidermis samples were obtained by peeling (*Arabidopsis*, radish, and cucumber) or scraping (wheat, rice, and maize) and observed under an inverted LM equipped with a color camera (**Figures 1** and **2** and **Figures S1D–F**). The boundaries of epidermis cells and the stomatal apparatuses are clearly outlined in all images (**Figures 1** and **2**). Compared with rice epidermises, the other plant epidermises were more easily obtained, and focusing of these epidermises was much better. Generally, the epidermis cells could be counted and measured manually or using a software package (Cheng et al., 2014).

Mesophyll cells are found in the layer underneath the epidermis. Except for guard cells, leaf epidermal cells do not contain a significant number of chloroplasts, which results in the epidermis being almost transparent. However, there were some green patches visible in the *Arabidopsis* (**Figure 1** and **Figure S1**), radish (**Figure 1**), wheat (**Figure 2** and **Figure S1**), and rice (**Figure 2**) epidermises. Mesophyll cells are tightly connected with epidermis cells in most of the samples tested, and they have been removed simultaneously with the epidermal cells or remained attached to the epidermis. It was thus hard to evenly separate mesophyll cells from the epidermis cells in some species when peeling or scraping leaf blades.

### Observation of Epidermises of Transparent Leaf Blade Under an LSCM

**Figure 3:**
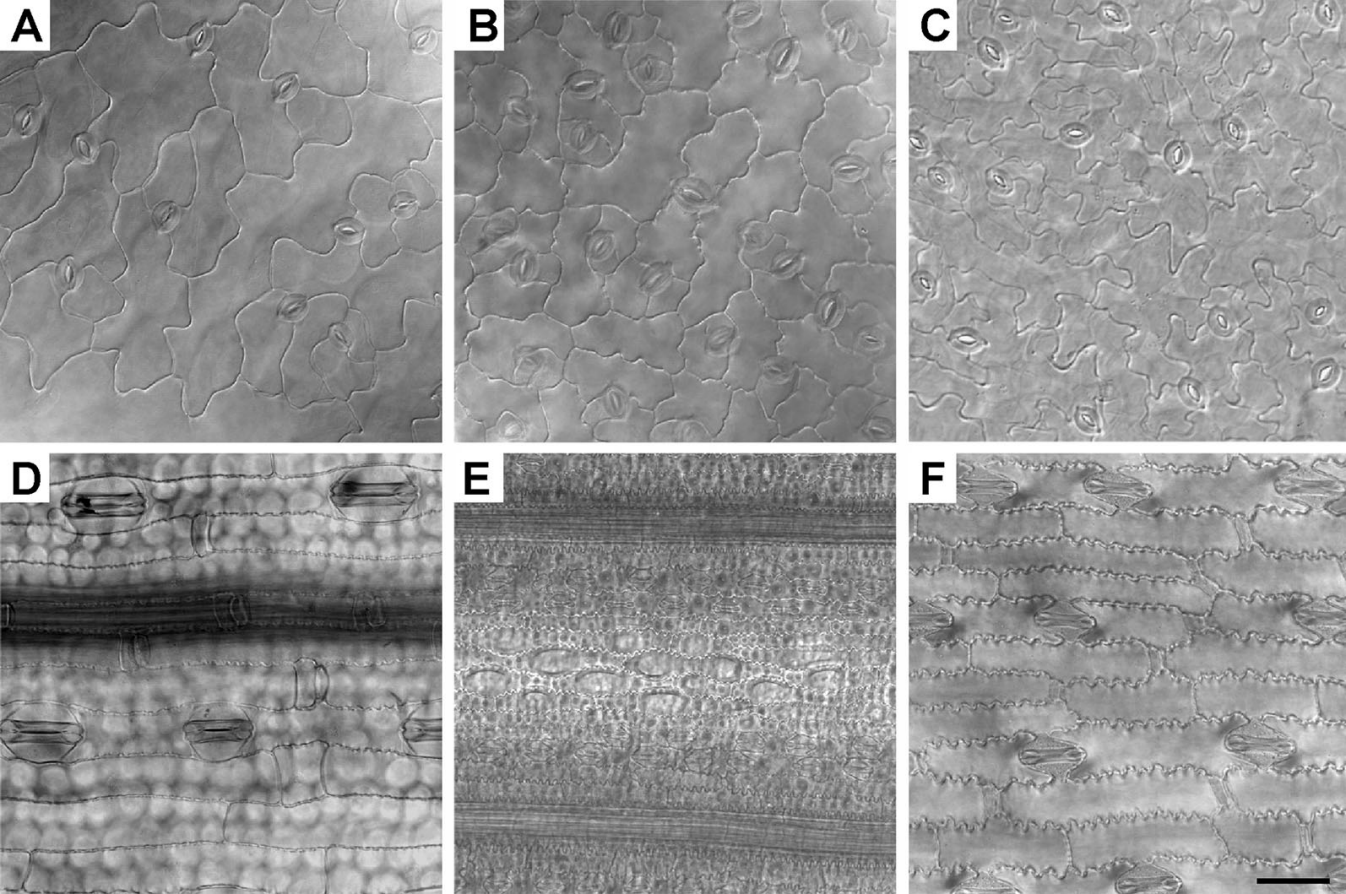
Epidermises of transparent leaf blades observed under an inverted LSCM. The leaf epidermises of *Arabidopsis* **(A)**, radish **(B)**, cucumber **(C)**, wheat **(D)**, rice **(E)**, and maize **(F)** were fixed, decolorized, and cleared. Bar=50 μm.

The isolated samples of whole leaf blades were cleared with a chloral hydrate mixture (Cheng et al., 2014) to obtain a transparent leaf blade sample before observation under an LSCM (in the DIC mode) (**Figure 3**). The objective lens of an LSCM of a certain magnification has a higher configuration when compared with an ordinary LM. For living cells and undyed specimens, it is easier to locate and identify transparent cells using the DIC mode under bright-field. We used the DIC mode to investigate leaf epidermises since the epidermises are almost transparent. This clearing method is convenient and only requires two steps: decolorizing and clearing. To extend the sample storage period, we modified this protocol; it has no obvious effects on the sampling quality (data not shown). It is generally known that DIC mode is better at investigating transparent cells than basic mode. Thus, the epidermis images of our transparent leaf blade samples were only investigated under an LSCM (in the DIC mode) but not under a basic LM that is not equipped with DIC modules.

Generally, in the pseudo 3D bright-field images (**Figures 3A–D, F**) captured with an LSCM using transmitted light, the cells were clearly shown with good resolution, which means the shortest distance between two points on a specimen of an optical microscope can still be distinguished by the observer or camera system as separate entities. Using this method, the LSCM images of all epidermises were well focused except those of the rice epidermis (**Figure 3**). Different parts of the rice epidermis had different focal planes. Only a small part of the intact rice epidermis was in focus (**Figure 3E**), whereas all regions of the epidermises of other plants were in focus at the same focal plane.

**Figure 4:**
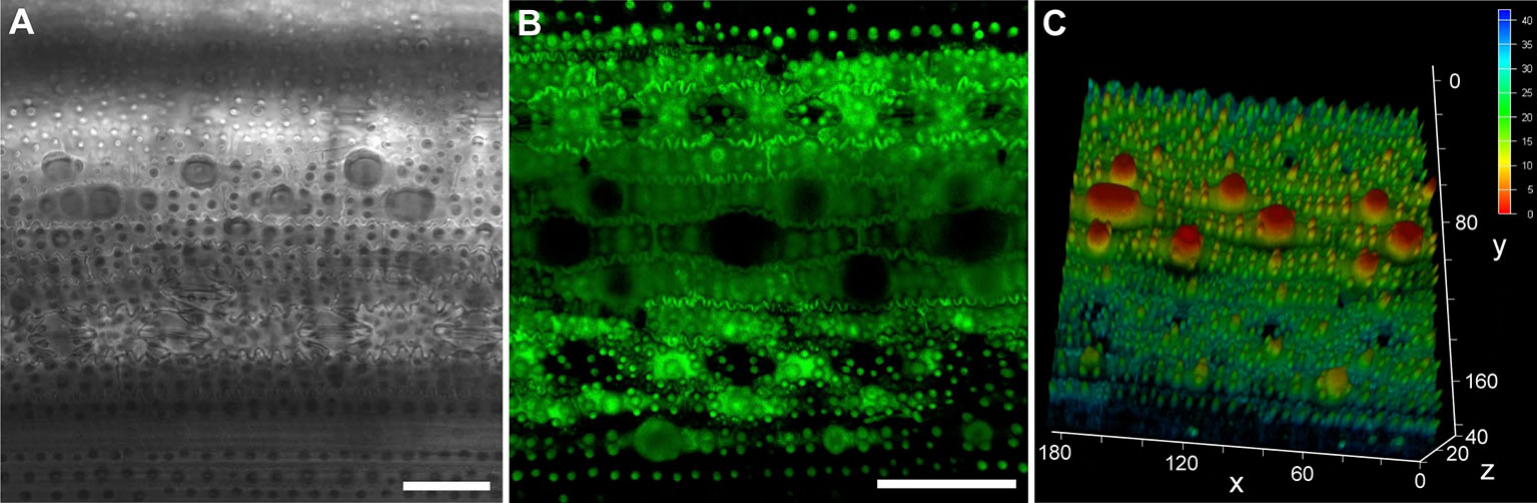
The epidermis of rice leaf blade under an LSCM. Bright-field image of chloral hydrate cleared leaf blade **(A)**. Fluorescence image of fresh leaf blade, two-dimensional **(B)**, and three-dimensional **(C)** structures are shown, respectively. Bar=50 μm, the values in **(C)** are in the unit of μm.

LSCMs are often used to detect the fluorescence of the cell surface and cell outlines (Wuyts et al., 2010; Jones et al., 2016; Januszkiewicz et al., 2019; Kierzkowski et al., 2019; Vaahtera et al., 2019; Zubairova et al., 2019). Although the bright-field images of chloral hydrated rice leaf tissues were only partially focused (**Figures 3E** and **4A**), rice tissue exhibited bright autofluorescence and the cell wall outlines could be clearly visualized (**Figure 4B**). In particular, a 3D surface structures of rice leaf blades could be obtained after compiling fluorescence signals of different Z layers (**Figure 4C**). Besides rice tissues, barley tissues also emitted autofluorescence (Talbot and White, 2013a). However, epidermis cell wall of *Arabidopsis* and cucumber leaf tissues showed little autofluorescence except the stoma, which exhibited bright autofluorescence (**Figure S2**).

### Observation of Leaf Epidermises Under an SEM

**Figure 5:**
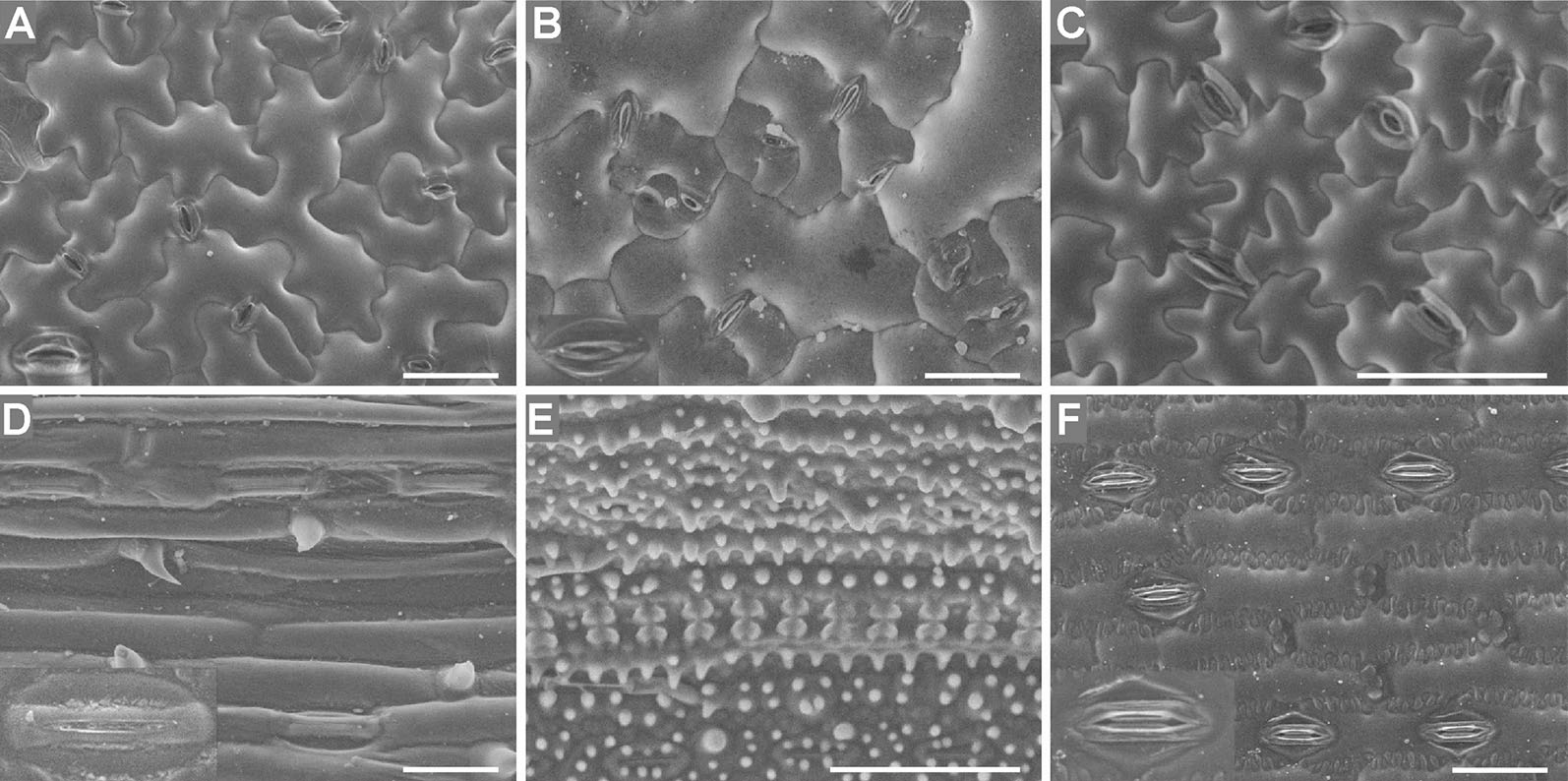
Fresh leaf epidermises observed under an SEM equipped with a Coolstage. Images of *Arabidopsis* **(A)**, radish **(B)**, cucumber **(C)**, wheat **(D)**, rice **(E)**, and maize **(F)** epidermises are shown. Enlarged images (2×) of stomatal apparatuses are shown in the lower left corners for all plants except cucumber and rice. Bar=50 μm.

Both the Coolstage method and the conventional method were used to investigate the micro-morphology of the leaf epidermises. The images in **Figure 5** were of fresh samples taken under an SEM with a Coolstage. Using the SEM Coolstage method, epidermis cells (not including stomatal apparatuses) generally appeared full and had clear outlines, with no obvious wrinkles, cracking, or collapse on the surfaces of the cells (**Figure 5A**), and epidermis cells at different depths in the same field were all in focus. However, some leaf samples were not stable over time under the Coolstage. For example, *Arabidopsis* leaf epidermis cells on the SEM Coolstage collapsed within about ten min after illumination (**Figure S3**), and the stomatal apparatuses were particularly prone to collapse. Thus, it would be very hard to take a complete image and to explore different parts of the sample. The stomatal apparatuses of *Arabidopsis*, radish, cucumber, and maize were deformed and not very clear (**Figures 5A–C, F**).

**Figure 6:**
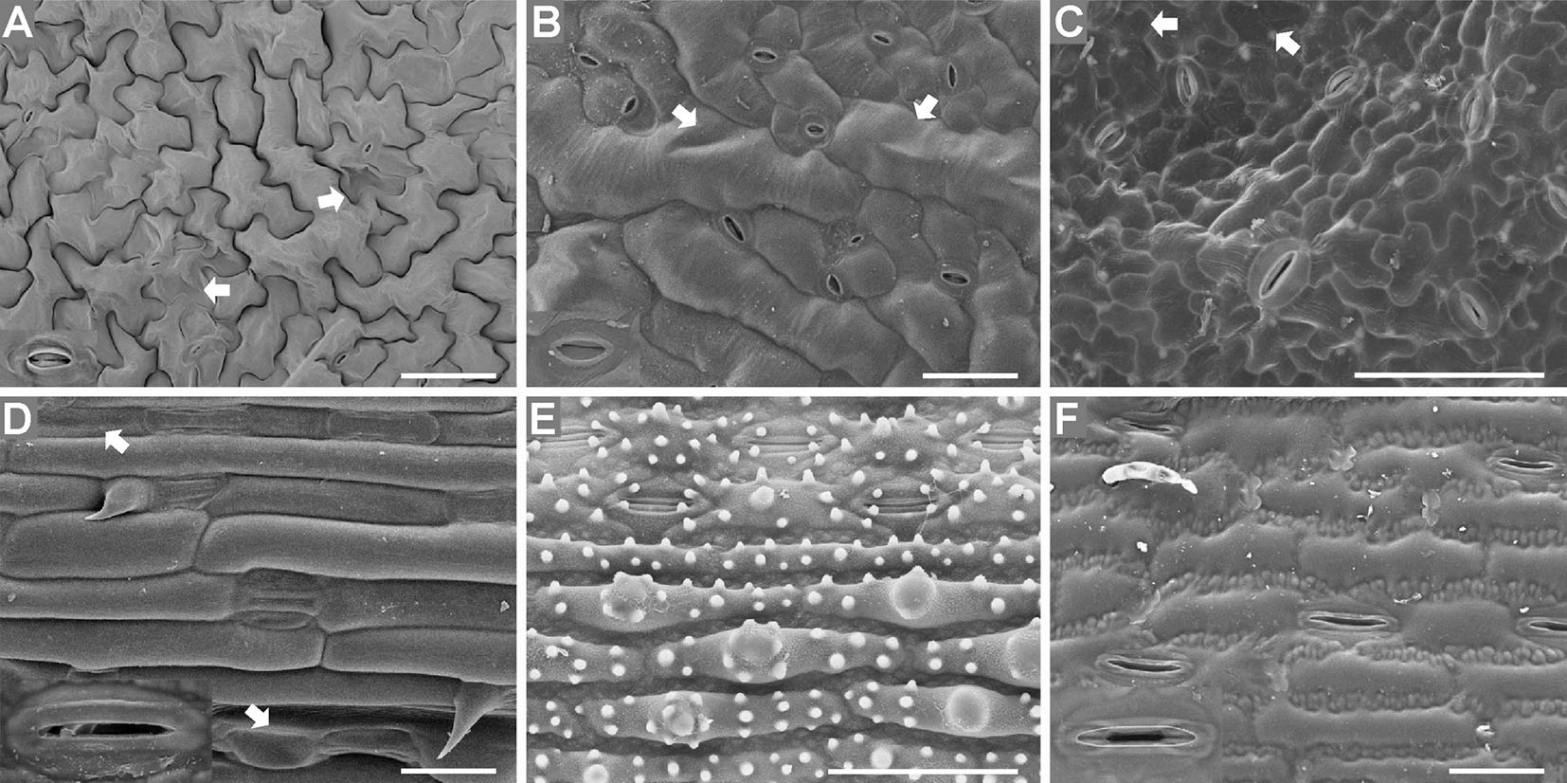
T-butanol freeze-dried, gold-coated leaf epidermises under an SEM. Images of *Arabidopsis* **(A)**, radish **(B)**, cucumber **(C)**, wheat **(D)**, rice **(E)**, and maize **(F)** epidermises are shown. Enlarged images (2×) of stomatal apparatuses are shown in the lower left corners for all plants except cucumber and rice. Arrows illustrate the local deformation of some epidermal cells. Bar=50 μm.

The images in **Figure 6** were taken under an SEM with a standard sample stage of samples prepared using the conventional method. Local deformation of some epidermal cells was induced by this method, and this deformation varied between species, which had different leaf water contents (**Figure 6**). The water contents of *Arabidopsis* (89.5% ± 0.4), radish (89.5% ± 0.5), cucumber (88.3% ± 0.5), and wheat (84.4 ± 0.3%) leaves were higher than those of rice (68.7 ± 1.0%) and maize (66.8 ± 1.1%) leaves, and locally deformation of some epidermis cells was observed in the leaves with high water contents prepared using the conventional method (**Figures 6A–D**). No deformation was observed in rice or maize leaves (with low water contents) (**Figures 6E, F**).

### Comparison of Different Preparation and Imaging Methods for Investigating the Micro-Morphology of the Leaf Epidermis

A comparison of the four different preparation and imaging methods is shown in **Table 1**. Regarding the preparation time and vision effect, there were obvious differences among images captured using different microscope configurations: basic LM, LSCM (in the DIC mode), SEM equipped with Coolstage, and SEM equipped with a standard sample stage (**Table 1**). In general, the preparation of all samples was fast, convenient, or simple, except for the preparation of the conventional SEM samples, which was complicated and time-consuming. When comparing images, the resolution of images taken under a basic LM was found to be low (**Figures 1** and **2**) while that under an SEM equipped with a standard sample stage was high (**Figure 6**). Compared with bright-field images captured using LM and LSCM (**Figures 1**–**3**), the SEM images were clearer and had a stereoscopic effect (**Figures 5** and **6**).

**Table 1:** Comparison of different preparation and imaging methods for investigating the micro-morphology of the leaf epidermis.

Sample preparation method	Preparation time	Imaging method	Image vision effect	Status of stomatal apparatus	Shortcomings	Merits
Direct epidermis isolation method	About 2 min	Inverted microscope equipped with color camera	1. Flat image and low resolution 2. All epidermis cells were in focus	Flat	Requires skill and patience; need to avoid main veins when preparing the specimen	1. No pre-treatment and short preparation time 2. The whole specimen was well focused at different cell layers
Chloral hydrate clearing method	One to several days	Inverted LSCM, DIC mode	1. Epidermises of all species were in focus except rice, where only some of the cells were in focus 2. Embossed effect	Embossed cell boundaries	Not suitable for observing leaf epidermises (such as rice epidermises) under bright-field, whose surfaces are largely undulated and hard	1. Flexible treatment time 2. Short working time
SEM Coolstage method	Less than one minute	SEM equipped with Coolstage, low acceleration voltage (5 kV), −15°C	1. The full shapes of epidermis cells were well maintained 2. Stereoscopic effect	1. Locally deformed in some species, e.g., *Arabidopsis* 2. Deformation was not obvious in wheat and rice, but resolution was low	1. Lower resolution compared with conventional SEM 2. Leaf epidermis cells of plants, e.g., *Arabidopsis*, deformed within about 10 min due to water loss under high vacuum	1. No pre-treatment and short preparation time 2. Fresh tissues observed directly, original appearance well maintained 3.Suitable for observing undulated tissues
Conventional SEM t-butanol freeze-drying method	Two days	SEM equipped with standard sample stage, high acceleration voltage (15 kV), but 5 kV was used for *Arabidopsis*	1. Some epidermis cells were locally deformed due to treatment 2. Stereoscopic effect and high resolution	1. Stereoscopic effect 2. High resolution and magnification	1. Specimen preparation is time-consuming and complicated, need equipment for specimen drying and metal coating 2. Improper operation during the drying process easily results in cell deformation	1. High resolution 2. Specimen stable under high vacuum 3. Suitable for observing undulated epidermises

Regarding the LM methods, we found that almost all parts of the visual field were focused under an inverted basic LM (equipped with a color camera) for samples prepared using the direct epidermis isolation method. However, only part of the sample was well focused using the LSCM. To illustrate it, images of *Arabidopsis*, cucumber, and wheat leaves taken under an inverted LM and those taken under an LSCM are shown (**Figure S1**). Moreover, the scrapped epidermises of rice were more difficult to focus especially when observed under the high magnification (**Figure 2E**) compared with other species (**Figures 1** and **2**). As for the chloral hydrate clearing method, the near-transparent leaf samples of rice were only partly in focus under an LSCM (**Figure 3E**), but in the SEM images of rice, the epidermises were well focused with a large depth of field (**Figures 5E** and **6E**).

Regarding the SEM methods, the shape and appearance of epidermis cells except stomatal apparatuses were more natural and maintained better using the SEM Coolstage method (**Figure 5**) compared with the conventional t-butanol freeze-drying method (**Figure 6**); however, local deformation due to the sample preparation treatment was found in the conventional SEM leaf samples with high water contents (**Figures 6A–D**). Specimens using the conventional SEM method could be observed under much higher magnification and were more stable under high vacuum. In contrast, the fresh sample of *Arabidopsis* was not stable in the SEM Coolstage method and deformation of cells of stomatal apparatuses occurred in some species (**Figures 5A–C, F**). Moreover, at the same magnification, the image resolution (related to the ability to observe fine details) was higher when using the conventional method (**Figure 6**) compared with the Coolstage method (**Figure 5**).

### The Effect of SEM Methods on Tissue Dimension, Stability, and Cell Morphology

**Figure 7:**
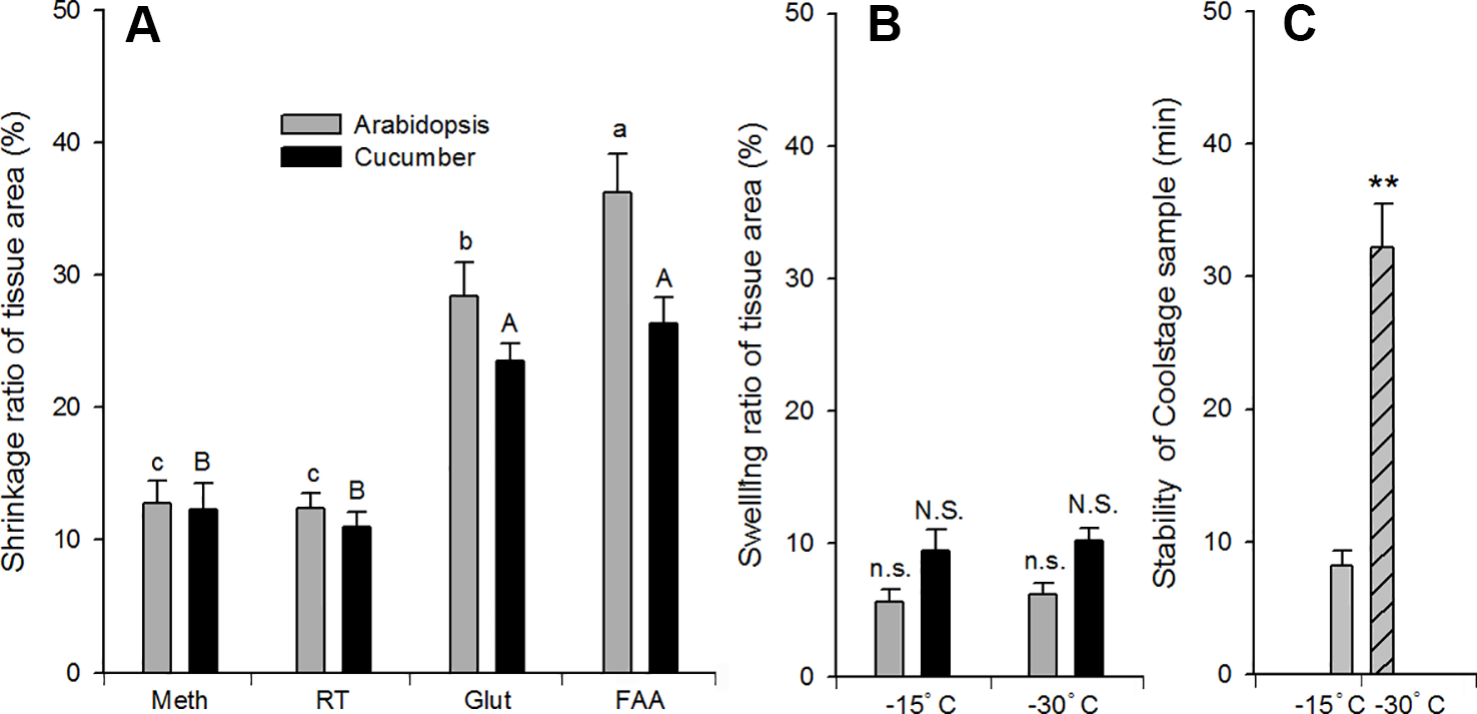
Effect of SEM methods on dimension and sample stability of *Arabidopsis* (
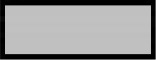
 and 
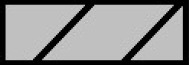
), cucumber (
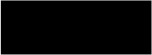
) leaf tissue. Shrinkage **(A)** or swelling **(B)** ratio after processing is expressed as percentage of original (fresh) tissue area. Different SEM procedures prior to t-butanol freeze-drying in **(A)**: meth, Glut, FAA = methanol, glutaraldehyde, and FAA fixation, respectively, followed by a graded series of both ethanol (on ice) and t-butanol; RT = methanol fixation and ethanol dehydration at room temperature, followed by a graded series of t-butanol (details in text). Treatments in **(B)**: −15°C = samples attached to double sided sticky carbon tabs on the Coolstage at −15°C; −30°C = samples on top of optimal cutting temperature compound in the Coolstage at −30°C. Values are means of 6–12 replications; vertical bars indicate ± SE. Different letters in **(A)** indicate significant differences among treatments at *P* < 0.05 based on Duncan’s new multiple range test and the data of **(B, C)** were analyzed by t-test. Double stars (**) indicate extremely significant difference (*P* < 0.01).

The tissues were cut into pieces 3∼5 mm^2^ and curly areas were avoided. The shrinkage ratio of *Arabidopsis* and cucumber tissues responded similarly to different SEM sample preparation methods. FAA or glutaraldehyde fixation resulted in an obvious shrinkage (24∼36%, *P* < 0.05) compared with methods using methanol as the fixative (8∼12%) (**Figure 7A**). In *Arabidopsis*, FAA fixation resulted in a more obvious shrinkage than glutaraldehyde fixation. With respect to the swelling ratio, there was no obvious difference between the two Coolstage methods (**Figure 7B**). The Coolstage methods resulted in a 6∼12% expansion of leaf dimensions. However, if samples were placed in OCT compound and observed at a lower temperature (−30°C), the stability of samples was significantly increased from an average of 8 min (on carbon double side stick tabs and at −15°C) to about 32 min (*P* < 0.01) (**Figure 7C**).

**Figure 8:**
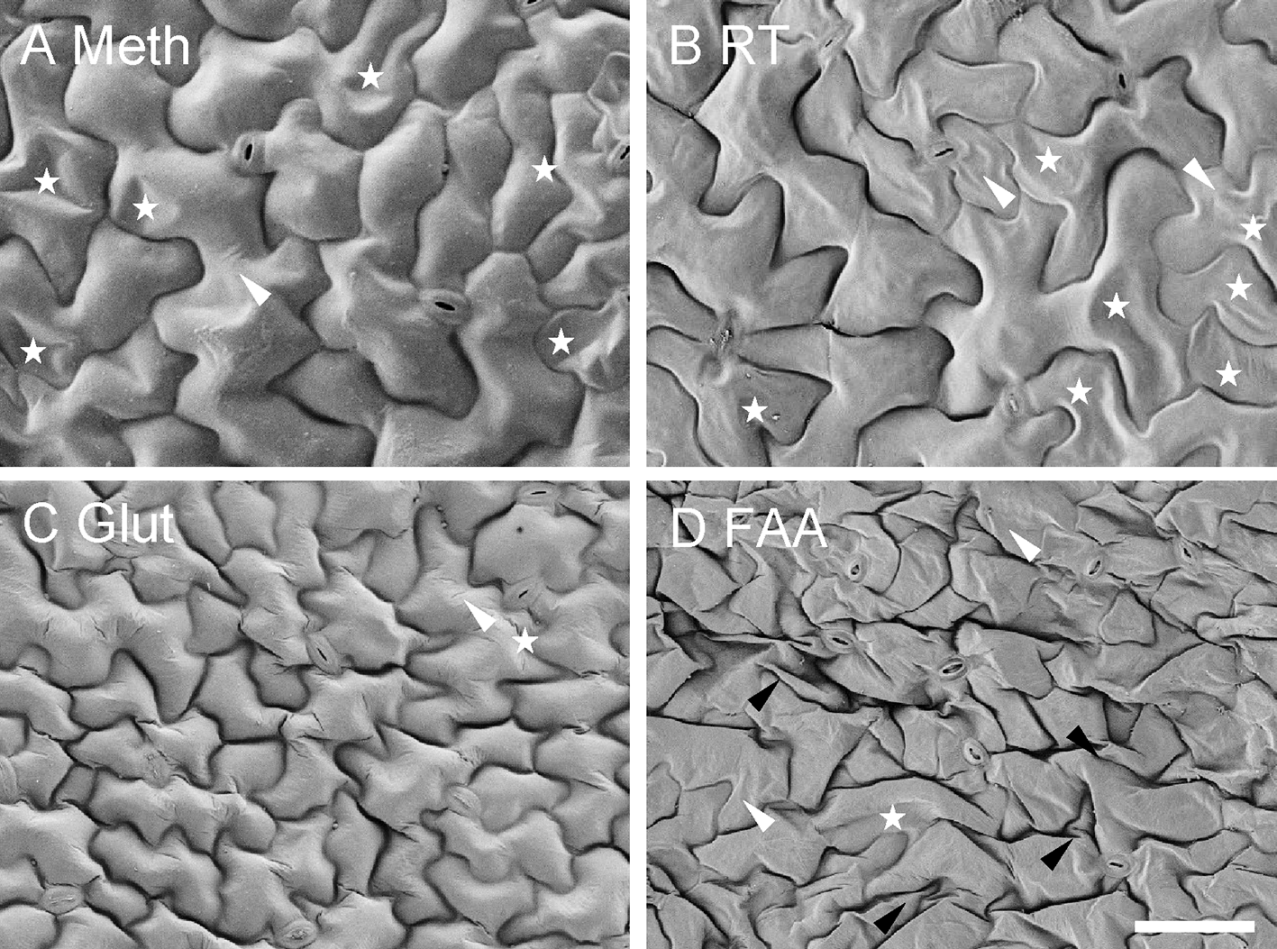
Effect of different SEM methods on morphology of *Arabidopsis* leaf epidermal cells. Each method includes a final step of t-butanol freeze-drying. **(A, C, D)** Meth, Glut, FAA = methanol, glutaraldehyde, and FAA fixation, respectively, followed by a graded series of both ethanol (on ice) and t-butanol; **(B)** RT = methanol fixation and ethanol dehydration at room temperature, followed by a graded series of t-butanol (details in text). Stars indicate partial cell collapse, black arrowheads show cell wall folding, and white arrowheads indicate cell wall wrinkles. All images are at the same magnification. Scale bar = 50 μm.

There was no obvious difference between the two Coolstage methods as related to tissue morphology (data not shown). The effects of four different SEM methods on tissue morphology were consistent with the effects on tissue dimensions (**Figure 8**). Generally, the cells seemed to be smaller (due to shrinkage) in the images using FAA or glutaraldehyde. However, cell shapes were well maintained in the images from method “Meth” and “Glut.” The cells of samples fixed with FAA were shrunk and deformed more than the samples fixed with glutaraldehyde, which was not surprising based on previous observations. The images from the method “Meth” seemed to be better than those from method “RT.” Our collective results demonstrate that a series of treatments were sometimes superior to a simple grade of treating.

## Discussion

Different preparation and imaging methods can affect the quality of leaf sample images (Kawai et al., 2013; Morawetz, 2013; Urban et al., 2018). Research related to methods for observing leaf micro-morphology provides alternative preparation and imaging methods, and improves work efficiency (Schwab and Hülskamp, 2010; Talbot and White, 2013a; Talbot and White, 2013b; Jayakody et al., 2017). However, the suitable microscope and preparation method for observing the micro-morphology of leaf epidermis samples is seldom studied. Here we compared different methods for preparing and imaging leaves of six species to provide some information for choosing methods for different species and applications.

First, we used a basic LM and an LSCM to observe leaf samples prepared using two different methods. Regarding the application of basic LM and LSCM (in the DIC mode) for investigating different leaf epidermis samples under bright-field, both microscopes had advantages. We found that the different parts of the direct isolated epidermises were focused better using the basic LM; however, the resolution of the images was higher using an LSCM (in the DIC mode). It was hard to focus on all parts of the epidermis in a single frame under the LSCM when observing the rice specimens under bright-field. That might be partly because the objective lens of LSCM has a higher NA (for example, 20× 0.75) than that of the basic LM (0.4), which results in a low focus depth of the LSCM. For some leaf samples, using a dye or changing the open degree of the condenser aperture diaphragm might also help to improve the resolution of LM samples. We also found that preparation of specimens using the direct isolation method required more skill and patience, although it seems fast and easy. The removal of most chloroplasts attached to the epidermises was achieved using KOH and hypochlorite as clearing or bleaching reagents after isolation. Additionally, making impressions of the leaf surface using either fingernail polish or 5% acrylic spray would be an alternative choice to avoid the influence of the mesophyll cells (Waisel et al., 1969; Gitz and Baker, 2009).

As for the chloral hydrate clearing method, the bright-field images of all epidermises were well focused under an LSCM except those of the rice epidermis. However, when detecting the fluorescence of leaf epidermis in rice, the LCSM showed better focus since its confocal capability was used to detect the autofluorescence emitted by cell walls (**Figures 4B, C**). Rice leaves have a naturally undulated and hard surface with a thick cell wall, cuticle, and silicon, making it more difficult to simultaneously focus on different areas using bright-field microscopes (**Figures 2E** and **3E**). To solve this problem, using the leaf replica method to observe rice epidermis is a good choice since it is quick, easy, and accurate (Kusumi, 2013). Unlike the peeled or scraped epidermis, different parts of the impressed samples have a high degree of homogeneity. This trait helps since different parts of the leaf epidermis could be focused better when mounted on a bright-field microscope. Furthermore, using extended-focus imaging helps to get a generally well-focused image with z-stacking and some software such as ImageJ, Fiji, and confocal software, etc. It is also a good choice to use an LSCM to investigate the leaf surface structures by detecting and compiling fluorescence signals of different Z layers (**Figure 4C**). Another alternative solution is to use an SEM since the rice epidermis can be well focused under an SEM (**Figures 5E** and **6E**).

Second, we compared the SEM images of fresh leaf samples captured under a Coolstage with those of samples prepared with the conventional method captured under a conventional stage. Leaves are often soft and tender, and epidermis cells of leaves with high water contents might locally deform using the conventional t-butanol freeze-drying method (**Figures 6A–D**). Using an SEM equipped with a Coolstage was good at maintaining sample integrity since the dehydration, substitution, and freeze-drying procedures were skipped. However, if high resolution of leaf epidermis cells and investigation of the shape of stomatal apparatuses are required, the Coolstage might not be suitable in some species because the stomatal apparatuses of some leaves were not clearly visible under a Coolstage (**Figures 5A–C, F**), and the image resolution was generally lower compared with conventional method (**Figures 5** and **6**). Moreover, *Arabidopsis* cells were unstable under the Coolstage (**Figure S3**); thus, images should be taken as soon as possible to get an intact image of the epidermis before collapse occurs as ice crystals in the sample sublimated with time. The water vapor may interfere with the signal, and thus the images were less well defined. It might be better to observe specimens under a conventional sample stage using the conventional preparation method (**Figures 6A–C, F**), or to observe fresh leaves with a cryo-SEM under a lower temperature or a more stable micro-environmental condition (Read and Jeffree, 1991).

In our study, the images of *Arabidopsis* taken under an optical microscope at 200× magnification and those under the SEM at about 300× were convenient for counting cell numbers. The magnification suitable for counting cells may vary in other species. The stomatal apparatus and epidermal cell numbers could be easily counted with the software package (Cheng et al., 2014). To measure the cell size, it is important to obtain images of cells with high contrast outlines. Images of uncoated leaf tissue obtained using a backscattered electron detector under low vacuum conditions showed high contrast of cell outlines, making them suitable for semi-automated analysis (Talbot and White, 2013a). Moreover, confocal fluorescence images are of high resolution and can yield higher-contrast cell outlines than DIC images (**Figure 4**), as long as the cell wall components could emit sufficient fluorescence. For species that emit little epidermis cell wall autofluorescence, such as *Arabidopsis* and cucumber, staining leaves with a fluorescence dye such as fluorescein diacetate, propidium iodide, and aniline blueS or using plasma-membrane localized GFP materials can help in the detection of cell wall outlines (Talbot and White, 2013a; Li et al., 2015). Furthermore, progress of recent studies on the workflow and analytical tools for obtaining and processing the 3D confocal images contribute to the measurement of epidermal cell sizes (Wuyts et al., 2010; Kierzkowski et al., 2019; Zubairova et al., 2019).

Third, we compared different SEM methods and made some improvements of the t-butanol freeze-drying and Coolstage methods. Our studies support the idea that freeze-drying with the t-butanol method is a valuable alternative method to the CPD methods. This observation was consistent with previous studies which suggested that t-butanol is equivalent or sometimes superior to CPD (Inoué and Osatake, 1988; Baskin et al., 2014). However, when using vacuum freeze-drying from t-butanol, the pump oil needs to be changed more frequently since t-butanol damages the pump oil. Our results indicate that using methanol as a fixative could reduce the shrinkage of leaf tissue compared with glutaraldehyde and FAA. However, since every sample is unique, the appropriate steps and the duration of each step may vary. Tender and soft tissue might need shorter and gentler treatment. Our studies also indicate that the stability of samples on the Coolstage could be highly improved by putting samples in OCT compound and at a lower temperature; at lower temperatures, the OCT compound could provide solid support to sample as ice crystals in leaf sample sublimate with time under high vacuum.

## Conclusions

For some plants with similar leaf characteristics, our results can provide some useful information to researchers about experimental design and operation. The use of appropriate methods and microscope for the sample of interest might allow the micro-morphology of leaf epidermises to be more efficiently and accurately investigated. However, imaging effect might vary with plant species due to different leaf structure and composition.

## Data Availability Statement

All data generated or analyzed during this study are included in the article/**Supplementary Material**.

## Author Contributions

JY contributed to design of the study, carried out all experiments and analysis and drafted the manuscript. XW, HZ, YL, JZ, SY, MW, MH, QZ and LL performed some of the experiments and image analysis. JL and ML analyzed images and revised the manuscript. All authors read and approved the final manuscript.

## Funding

This work was supported by the National Natural Science Foundation of China (31670317, U1504319 and 31970380), the Natural Science Foundation of Henan Province (182300410057), and the Science and Technology Project of Henan Province (182102110234).

## Conflict of Interest

The authors declare that the research was conducted in the absence of any commercial or financial relationships that could be construed as a potential conflict of interest.

## Abbreviations

SEM, scanning electron microscope; LSCM, laser scanning confocal microscope; LM, light microscope; DIC, differential interference contrast; CPD, critical point drying; OCT, optimum cutting temperature.
