# Supplementary material for: Comparison of Sample Preparation Techniques for Inspection of Leaf Epidermises Using Light Microscopy and Scanning Electronic Microscopy
Source: Front Plant Sci. 2020 Feb 25;11:133. doi: 10.3389/fpls.2020.00133 (PMC7052180; doi:10.3389/fpls.2020.00133)
Supplement: Supplementary file 1 [file DataSheet_1.doc]

Supplementary figures of

# Comparison of sample preparation techniques for inspection of leaf epidermises using light microscopy and scanning electronic microscopy

**Jinhong Yuan,** **Xiaoduan Wang, Huihui Zhou, Yulin Li, Jing Zhang, Shuxin Yu, Mengni Wang, Menghan Hao, Qian Zhao, Le Liu, Mingjun Li, and Junhua Li**


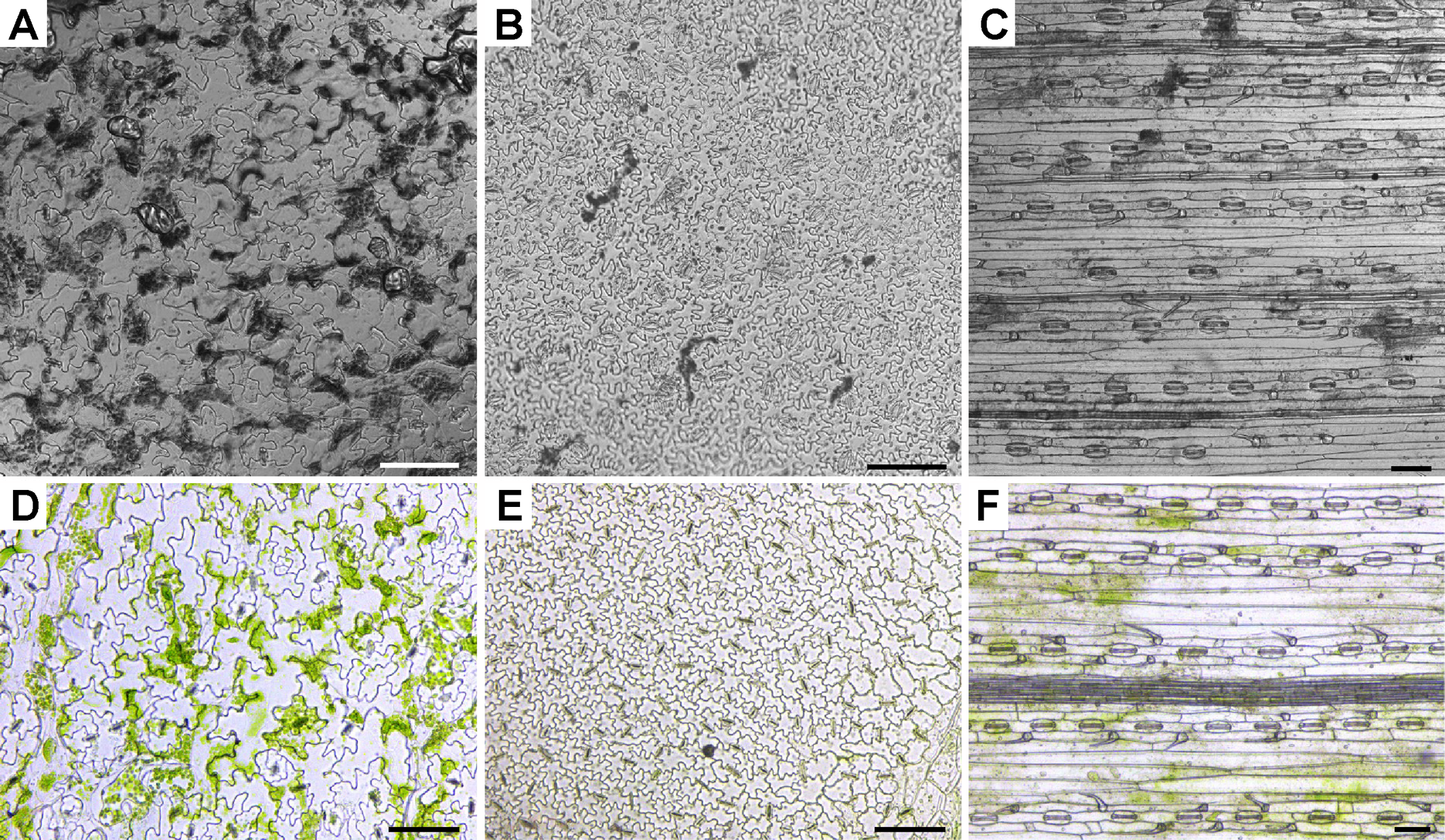


**Figure S1** Images of leaf epidermises taken under a basic LM or an LSCM. Images of the adaxial epidermises of Arabidopsis (A and D), cucumber (B and E), and wheat (C and F) are shown. The images A-C were taken under an LSCM, while images D-F were taken under a basic LM. Bar=100 μm.


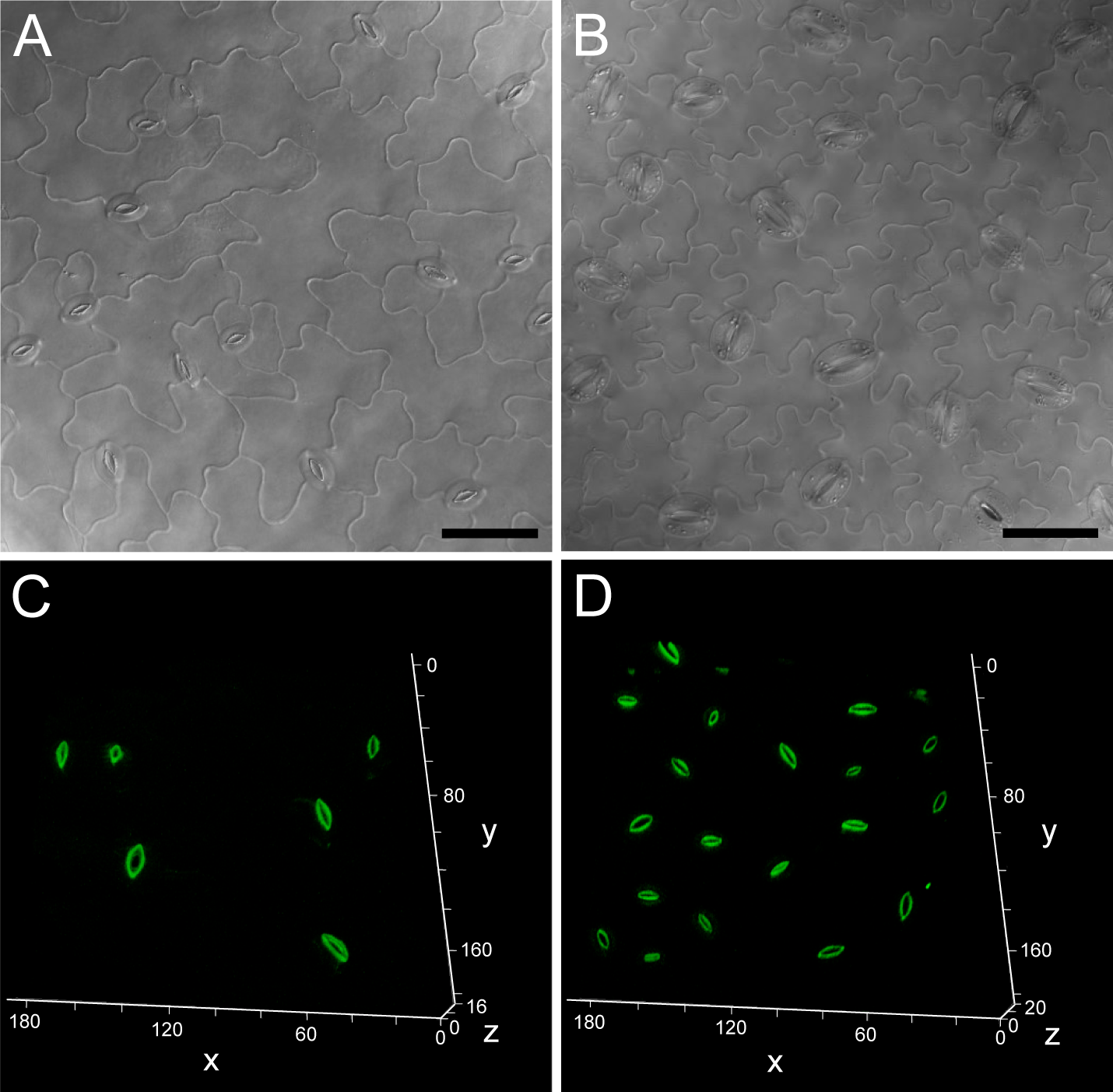


**Figure S2** The epidermises of Arabidopsis and cucumber leaf blades under an LSCM. Bright-field images of chloral hydrate cleared Arabidopsis and cucumber leaf blades (A and B, respectively). 3D fluorescence images of fresh Arabidopsis and cucumber leaf blades (C and D respectively). Bar=50 μm, the values in C and D are in the unit of μm.


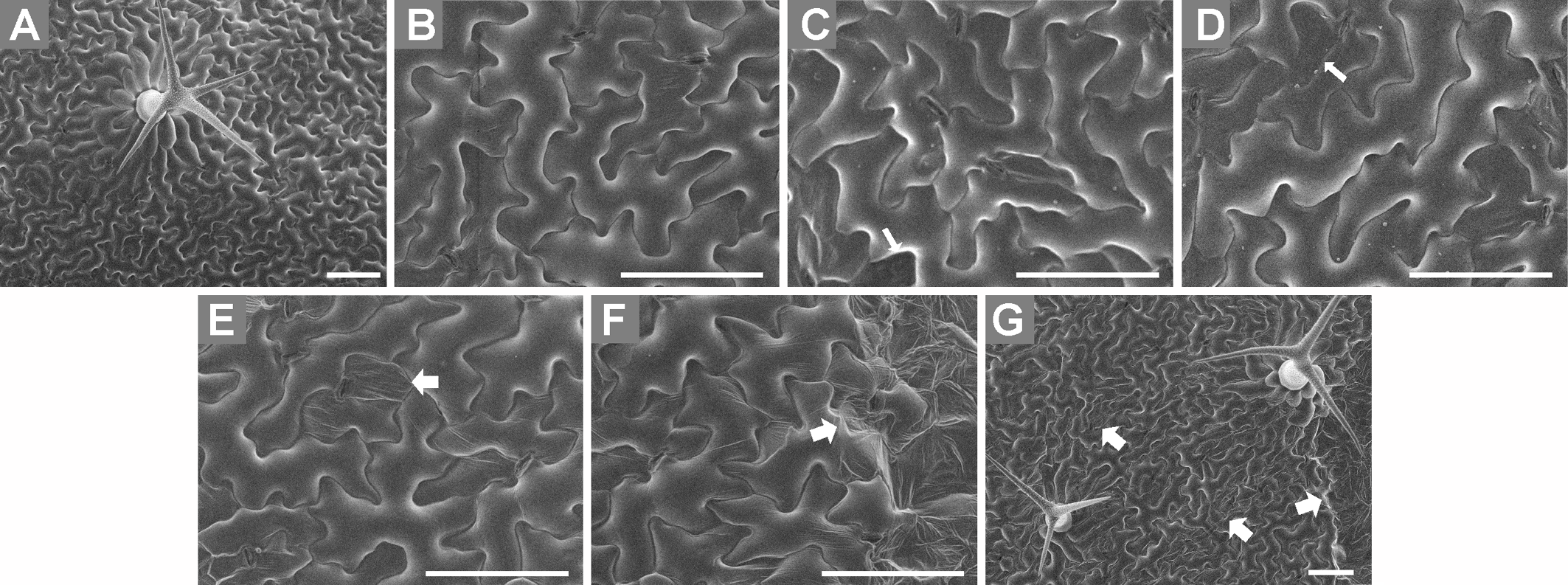


**Figure S3** The leaf cell shapes of Arabidopsis adaxial epidermis changed over time under an SEM Coolstage. Images A-G were taken 0, 2, 4, 6, 8, 10, and 12 min after illumination, respectively. Thin arrows show early collapse of the pressure-sensitive epidermis cells. Thick arrows show areas of deformation. Bar=100 μm.
